# Limited evidence of a shared genetic relationship between C-reactive protein levels and cognitive function in older UK adults of European ancestry

**DOI:** 10.3389/frdem.2023.1093223

**Published:** 2023-08-02

**Authors:** Amy Packer, Anne Corbett, Ryan Arathimos, Clive Ballard, Dag Aarsland, Adam Hampshire, Danai Dima, Byron Creese, Margherita Malanchini, Timothy R. Powell

**Affiliations:** ^1^Social, Genetic and Developmental Psychiatry Centre, Institute of Psychiatry, Psychology, and Neuroscience, King's College London, London, United Kingdom; ^2^College of Medicine & Health, St Luke's, University of Exeter, Exeter, United Kingdom; ^3^Department of Old Age Psychiatry, Institute of Psychiatry, Psychology and Neuroscience, King's College London, London, United Kingdom; ^4^Department of Brain Sciences, Faculty of Medicine, Imperial College London, London, United Kingdom; ^5^Department of Neuroimaging, Institute of Psychiatry, Psychology and Neuroscience, King's College London, London, United Kingdom; ^6^Department of Psychology, School of Health and Psychological Sciences, City, University of London, London, United Kingdom; ^7^Department of Clinical and Biomedical Sciences, University of Exeter Medical School, University of Exeter, Exeter, United Kingdom; ^8^Department of Biological and Experimental Psychology, School of Biological and Chemical Sciences, Queen Mary University of London, London, United Kingdom

**Keywords:** inflammation, C-reactive protein, cognitive function, polygenic risk score, aging, PROTECT study

## Abstract

**Introduction:**

Previous studies have shown associations between cognitive function and C-reactive protein (CRP) levels in older adults. Few studies have considered the extent to which a genetic predisposition for higher CRP levels contributes to this association.

**Methods:**

Data was analyzed from 7,817 UK participants aged >50 years as part of the PROTECT study, within which adults without dementia completed a comprehensive neuropsychological battery. We constructed a polygenic risk score (PRS-CRP) that explained 9.61% of the variance in serum CRP levels (*p* = 2.362 × 10^−7^) in an independent cohort. Regressions were used to explore the relationship between PRS-CRP and cognitive outcomes.

**Results:**

We found no significant associations between PRS-CRP and any cognitive measures in the sample overall. In older participants (>62 years), we observed a significant positive association between PRS-CRP and self-ordered search score (i.e., spatial working memory).

**Conclusion:**

Whilst our results indicate a weak positive relationship between PRS-CRP and spatial working memory that is specific to older adults, overall, there appears to be no strong effects of PRS-CRP on cognitive function.

## 1. Introduction

Marked inter-individual differences have been demonstrated in cognitive function and age-related decline (Foster, 2006). Understanding what causes this variability and identifying individuals at-risk of cognitive problems, is of critical importance to our aging population and workforce. Chronic low-grade inflammation, has been identified as one potential contributor to differences in brain age and cognitive function in older adults (Fard and Stough, 2019; Gordleeva et al., 2020).

C-reactive protein (CRP) is an acute-phase protein that has a critical role in the human immune system, and correlates with proinflammatory cytokine levels (Gabay and Kushner, 1999). Both CRP and cytokines found in blood can cross the blood-brain barrier to affect brain function (Quan and Banks, 2007). Animal studies have consistently shown an effect of inflammation on cognitive function. For example, administering the inflammatory agent lipopolysaccharide (LPS) to mice has been shown to cause learning and memory impairment (Salmani et al., 2020). LPS-induced inflammation impairs hippocampal neurogenesis in rats (Ekdahl et al., 2003), providing a putative mechanism connecting inflammation with brain function.

Elevated serum levels of CRP are considered a marker of systemic inflammation, and have been linked to several age-related conditions in humans, including cardiovascular disease, diabetes mellitus, and cognitive impairment (Cesari et al., 2003; De Rekeneire et al., 2006; Michaud et al., 2013). Higher CRP levels are also associated with reduced working memory (Bettcher et al., 2012); poorer executive function and processing speed (Tegeler et al., 2016); greater declines in visuospatial function (Warren et al., 2018); and smaller hippocampal volume across the lifespan (Wang et al., 2022). However, not all findings are consistent; discrepant findings may reflect differences in study design and sample characteristics, such as whether or not the participants being studied have an ongoing disorder (Carlier et al., 2021).

To date, few studies have investigated the relationship between genetic risk for CRP and cognitive function in typical aging. Those that have, are mostly lacking in statistical power or have focused on a limited number of individual CRP SNPs (~3 SNPs; for a review see, Stacey et al., 2017). For instance, one study used an unweighted PRS-CRP calculated using 47 GWAS significant SNPs and found that, after adjusting for sociodemographic and clinical confounders, contrary to hypotheses, higher PRS-CRP (and serum CRP) levels were significantly associated with better performance on a visuospatial memory test but not with reaction times (Milton et al., 2021). Investigation of PRS-CRP associations with a more comprehensive battery of cognitive tests is therefore warranted. Ultimately, if an association were confirmed, a PRS-CRP could be useful in prospectively identifying individuals at risk of both inflammatory conditions and poorer performance in specific cognitive tasks in adulthood. This would allow for the design of interventions such as exercise, anti-inflammatories, or brain training, to compensate for this predisposition (Bamidis et al., 2014; Melnikov et al., 2021).

We aimed to investigate the relationship between genetic risk for CRP levels and cognitive function in a sample of 7,817 individuals without dementia aged >50 years from the PROTECT study. Using the most powerful GWAS to date (i.e., N = 418,642; Han et al., 2020) we identified a PRS-CRP that explained 9.61% of the variance in serum CRP levels in an independent cohort (*N* = 268, age range = 20–84 years, females = 52.6%). We then assessed associations between PRS-CRP and performance on two cognitive batteries assessing a range of cognitive domains. Given evidence that CRP levels are negatively associated with cognitive function (Bettcher et al., 2012; Tegeler et al., 2016; Warren et al., 2018; Wang et al., 2022), we hypothesized that lower genetically predicted CRP levels would be related to better performance on the cognitive tests. Older individuals have the highest levels of CRP due to “inflammaging” (i.e., the age-related increase in systemic chronic inflammation, Fard and Stough, 2019; Gordleeva et al., 2020), and demonstrate more inter-individual variation in cognitive performance (see, Sánchez-Izquierdo and Fernández-Ballesteros, 2021), therefore we also used a median split to explore possible age-dependent associations specific to the oldest adults in our sample. Our results shed new and unexpected insights into the relationship between CRP levels and cognition in older adults.

## 2. Methods

### 2.1. Participants

Participants were recruited to the Platform for Research Online to investigate Genetics and Cognition in Aging study (PROTECT; http://www.protectstudy.org.uk/). PROTECT is a longitudinal UK-based online participant registry that aims to understand the impact of lifestyle, medical and genetic risk factors on cognitive health and dementia risk in older adults (Creese et al., 2021). At the time this project was conducted, the inclusion criteria for enrolling in PROTECT were (1) ≥50 years old; (2) no diagnosis of dementia; and (3) access to a computer and the internet. Volunteers were prospectively recruited from November 2015 through both local and national publicity. Data collection was ongoing at the time of this study; therefore, a data freeze was implemented in October 2019 with data extracted for analyses up to this date. The sample in the current study was a subset of PROTECT study participants who had completed cognitive testing, provided a saliva sample for genotyping, and were identified as individuals of European ancestry based on genetic principal components (PCs). We selected individuals of European ancestry to match the ancestry of the sample from which the serum CRP GWAS summary statistics were derived for construction of the polygenic risk scores (Han et al., 2020).

Ethical approval was granted through the London Bridge National Research Ethics Committee (reference: 13/LO/1578) and informed consent obtained for all participants. The authors assert that all procedures contributing to this work comply with the ethical standards of the relevant national and institutional committees on human experimentation and with the Declaration of Helsinki 1975, as revised in 2008.

### 2.2. Demographic, lifestyle, and medical data collection

We included the following demographic data, which are routinely collected during PROTECT, as covariates in our analyses: age, sex, education level (school until 16, school until 18, vocational qualification, undergraduate degree, post-graduate degree, and doctoral degree) and employment status (full time, part time, self-employed, retired, and unemployed). Covariate selection was informed by previous genetic research in the PROTECT sample (Creese et al., 2021).

### 2.3. Cognitive assessment

Cognitive performance was assessed using two online neuropsychological test batteries: the PROTECT Cognitive Test Battery (PCTB) and CogTrack^TM^. Each battery consisted of four tests from which a total of 12 outcome measures were derived and included in analyses (see, Table 1; or for a more detailed summary, Supplementary Table 1). Completion of the PCTB battery was mandatory, whereas the CogTrack^TM^ was optional. The PCTB takes approximately 10 min to complete and the CogTrack^TM^ takes approximately 15 min. Participants were instructed to perform the two test batteries up to three times over a period of seven days, leaving at least 24 h between each testing session. The tasks have parallel forms to ensure that repeated stimuli are not given to participants at each test session. Within PROTECT, it has previously been demonstrated that the use of three repeats substantially reduces within-person variability (Ballard et al., 2018).

**Table 1:** Summary of the neuropsychological test batteries and the derived outcome measures used in analyses.

**Battery test**	**Outcome measures**	**Cognitive domain assessed**
**PROTECT Cognitive Test Battery (PCTB)**		
Paired associates learning	Summary score	Visual-spatial working memory and learning
Digit span	Summary score	Working memory
Self-ordered search	Summary score	Executive function, spatial working memory
Verbal reasoning	Summary score	Verbal reasoning
**CogTrack** ^*TM*^		
Delayed Visual Recognition	Target picture accuracy (%) Distractor picture accuracy (%)	Visual memory Visual memory
Simple Reaction Time	RT (median)	Processing speed
Digit vigilance	RT (median) Accuracy (%) False alarms (n)	Processing speed Attention Attention
Choice Reaction Time	RT (median) Accuracy (%)	Processing speed Attention

RT, reaction time.

### 2.4. PROTECT genetic data

Saliva samples were collected by post and DNA was extracted by the National Institute for Health Research South London and the Maudsley National Health Service Biomedical Research Center. Genotyping was completed using the Illumina Global Screening Array with custom content (Illumina, California, USA).

The total number of participants in the genotyped data for the whole PROTECT study was 9,146. Genotype quality control (QC) was performed on all 9,146 individuals, as described in Creese et al. (2021). The QC involved iterative filtering for a call rate at 98% completeness (for individuals and SNPs) and then removing participants that were either related, of non-European ancestry based on genetic PCs, of mismatched sex, outliers in the PC calculation or detected to have excess heterozygosity. This resulted in the exclusion of 84 samples due to incompleteness and the removal of a further 794 individuals following further exclusions. Thus, a sample size of 8,268 participants remained.

Genotypes were imputed to 1,000 Genomes European reference panel using the Michigan imputation server and genotype phased using Eagle (Loh et al., 2016). Variants were restricted to SNPs only, with a MAF > 0.001. An absolute cut-off of 0.7 was applied to the imputation quality of variants (Rsq as reported by the Michigan imputation server). The number of variants remaining after quality control was 9,415,055.

Further genotype QC was performed within the sample of 8,268 individuals of European ancestry, following all exclusions. The following SNP exclusions were applied, minor allele frequency (MAF) of <1% and those not in Hardy-Weinberg Equilibrium (*p*-value < 0.00001).

### 2.5. Polygenic scores for C-reactive protein

To calculate polygenic risk scores for C-reactive protein levels (PRS-CRP) we used genome-wide summary statistics (clumped using 250 kb windows and *r*^2^ > 0.1) from Han and colleagues who performed the largest GWAS of serum C-reactive protein levels to date (*N* = 418,642 participants of European ancestry aged 40–69 years from UK Biobank; Han et al., 2020). Using these summary statistics, we defined the optimal *p*-value threshold (*P*_*T*_) for polygenic risk for serum CRP levels in an independent cohort (i.e., the *P*_*T*_ explaining the most variance in CRP levels). We then calculated a PRS-CRP in the PROTECT sample, using the optimal *P*_*T*_ we had identified. We used PRSice-2 version 2.3.3 software (Choi and O'Reilly, 2019) to identify the optimal *P*_*T*_ and calculate all PRS-CRP.

#### 2.5.1. Defining the optimal p-value threshold in an independent cohort

We used data from the South East London Community Health Study (SELCoH; Hatch et al., 2011) to define the optimal *P*_T_ for PRS-CRP.

##### 2.5.1.1. Participants (SELCoH cohort)

We used data from self-reported white individuals, previously confirmed to be of European ancestry (see, Powell et al., 2021; N = 268, aged 20–84 years). We selected individuals of European ancestry to match the ancestry of both the current PROTECT sample and the sample from which the serum CRP GWAS summary statistics were derived (i.e., Han et al., 2020). In sensitivity analyses, we further divided this subsample into four equal sized groups based on age (*n* = 67 per group).

The SELCoH study received ethics approval from the King's College London research ethics committee, reference PNM/12/13-152. All participants provided written informed consent to taking part in the study.

##### 2.5.1.2. Serum CRP level

Serum levels (pg mL^−1^) of CRP (and other blood-based markers) were assessed in blood samples from the SELCoH cohort using multiplex ELISA-based technology provided by the Meso Scale Discovery Biomarker kits, as described previously (Powell et al., 2020, 2021; Palmos et al., 2021). DNA samples from the SELCoH cohort were sent to the Affymetrix Research Services Laboratory in Santa Clara, CA, USA.

##### 2.5.1.3. SELCoH genetic data

Genotyping was assayed using the UK Biobank Axiom Array (r3) which comprises of 820,967 genetic markers (Affymetrix, Santa Clara, CA, USA). Genotype imputation was performed on the Michigan Imputation Server v1.2.4 (https://imputationserver.sph.umich.edu/index.html), using Eagle v2.4 (Loh et al., 2016) phasing and the 1,000 Genomes Phase 3 v5 (mixed population) as a reference panel. Genotype data underwent standard quality control procedures as described previously (Powell et al., 2020; Palmos et al., 2021).

##### 2.5.1.4. Optimal *p-*value threshold

We used PRSice-2 to determine the optimal *p*-value threshold (*P*_*T*_) for the PRS-CRP calculated from the GWAS summary statistics (Han et al., 2020). The CRP data was log-transformed and adjusted for assay batch, age, sex, BMI, and smoking. This CRP phenotype was then modeled in PRSice-2 with the first seven ancestry population covariates (PCs) as covariates (to adjust for population structure in the SELCoH sample, Patterson et al., 2006; Price et al., 2006), for *p*-value thresholds from *p* = 0.001 to *p* = 0.500, increasing in 0.001 increments. Bonferroni correction was applied to account for the 500 thresholds tested.

### 2.6. Statistical analyses

All statistical analyses were performed using RStudio version 1.3.1093 and R version 4.0.3. Only participants with both cognitive and genotype data from the PROTECT sample were included in the analyses. All available data from the PCTB and CogTrack^TM^ were averaged (mean) across participants' completed test sessions to obtain a baseline measurement for each cognitive outcome. PRS-CRP were standardized to a mean of 0 and standard deviation of 1 before analysis. Regression coefficients thus represent a unit increase in cognitive composite score per one standard deviation increase in PRS-CRP. Separate multiple linear regressions were performed with each of the cognitive outcomes (Table 1) as dependent variables, with PRS-CRP as the independent variable, age, sex, education level (school until 16, school until 18, vocational qualification, undergraduate degree, post-graduate degree, and doctoral degree), employment status (full time, part time, self-employed, retired, and unemployed), number of test sessions completed, and first six ancestry PCs fitted as covariates simultaneously. Dummy variables were created for all variables with ordinal and categorical responses. The proportion of variance explained by the PRS-CRP is represented by the *R*^2^ of the model with only covariates subtracted from the *R*^2^ of the full model. Covariate selection (demographic and population structure) was informed by previous research in this sample investigating the effects of a PRS for Alzheimer's disease (Spellman et al., 2015). The number of PCs included as covariates differs to that used with the SELCoH cohort, reflecting the different numbers needed to account for population structure, as assessed via PC scatter plots. Bonferroni correction for multiple testing was applied (i.e., *p* < 0.004; α = 0.05/12, to reflect the number of outcome variables). Outliers of more than +/– 2 standard deviations from the mean (adjusting for phenotypic covariates) were removed from all cognitive outcome variables.

### 2.7. Sensitivity analyses

#### 2.7.1. Variance explained by PRS-CRP at different ages

As the mean age of the SELCoH cohort (mean = 49.82) was younger than that of PROTECT (mean = 62.48), we aimed to determine whether the polygenic risk score generated in the SELCoH sample (*n* = 268), explained similar amounts of variance in different age bins (*n* = 67; ages 20–38, 39–49, 49–62, 62–84). We performed separate linear regressions within each age bin to test the amount of variance explained by PRS-CRP (adjusted for the first 7 PCs) on log-transformed serum CRP levels (adjusted for age, gender, BMI, assay run, and smoking).

#### 2.7.2. Relationship between PRS-CRP and cognition at different ages

To assess whether our PRS-CRP differentially predicts cognition in middle and older age adults, we repeated analyses in the PROTECT sample using a median split based on age (i.e., 62 years for both PCTB and CogTrack^TM^). A median split was used so that the sample size and power of each group were approximately equal.

## 3. Results

### 3.1. PROTECT participant characteristics

Between October 2015 and May 2018, 7,817 participants with genetic data completed the mandatory PROTECT cognitive battery and 7,275 (93.10%) also completed the optional the CogTrack^TM^ battery within 2 months of having completed the PROTECT battery. Participant characteristics are shown in Table 2. Descriptive statistics for each cognitive outcome variable are shown in Table 3. Outliers and missing data are summarized in Supplementary Table 2.

**Table 2:** PROTECT participant characteristics (*N* = 7,817).

**Variable**	**PCTB (*****n*** = **7,817)**		**CogTrack**^**TM**^ **(*****n*** = **7,275)**	
Age (mean, SD)	62.48	7.17	62.51	7.19
**Sex (** ***n*** **, %)**				
Male	1,959	25.06	1,860	25.57
Female	5,858	74.94	5,415	74.43
**Education level (n, %)**				
Secondary education	1,180	15.10	1,074	14.76
Post-secondary education	922	11.79	1,198	11.75
Vocational qualification	1,569	20.07	1,456	20.01
Undergraduate degree	2,624	33.57	2,451	33.69
Post graduate degree	1,276	16.32	1,198	16.47
Doctoral degree	246	3.15	241	3.31
**Employment status (n, %)**				
Full-time	1,402	17.94	1,283	17.64
Part-time	1,324	16.94	1,232	16.93
Self-employed	814	10.41	760	10.45
Retired	4,047	51.77	3,787	52.05
Unemployed	230	2.94	213	2.93
**Test sessions completed (** ***n*** **, %)**				
1	566	7.24	822	11.30
2	1,123	14.37	1,640	22.54
3	6,128	78.39	4,813	66.16

PCTB, PROTECT cognitive test battery; SD, standard deviation.

**Table 3:** Descriptive statistics for the cognitive outcomes.

**Test battery Cognitive outcome**	***n***	***M***	***SD***	**Min**	**Max**
**PCTB**					
Paired associates learning	7,469	4.51	0.63	2.50	6.33
Digit span test	7,553	7.31	1.20	3.67	10.67
Self-ordered search	7,406	7.79	1.70	2.00	12.67
Verbal reasoning	7,464	31.98	7.93	3.00	56.00
**CogTrack** ^*TM*^					
Delayed visual recognition sxaccuracy (original image)	6,932	91.09	6.72	15.00	100.00
Delayed visual recognition accuracy (distractor image)	6,978	69.39	15.67	0.00	100.00
Simple Reaction Time Speed (median)	6,959	339.85	49.95	226.73	993.90
Digit Vigilance Speed	7,081	485.70	41.41	352.33	843.00
Digit Vigilance Target Accuracy	7,105	98.39	1.97	87.78	100.00
Digit Vigilance False Positive Responses	6,990	1.65	1.24	0.00	6.50
Choice Reaction Time (median)	7,062	511.38	50.36	375.50	690.50
Choice Reaction Time Accuracy	7,009	97.69	1.72	92.00	100.00

PCTB, PROTECT cognitive test battery; *n* = sample size accounting for missing data and following the removal of outliers (missing data and outliers are summarized in Supplementary Table 2); *M*, mean; *SD*, standard deviation. Outliers +/– 2 standard deviations from the mean (adjusting for covariates) have been removed.

### 3.2. PRS-CRP

**Figure 1:**
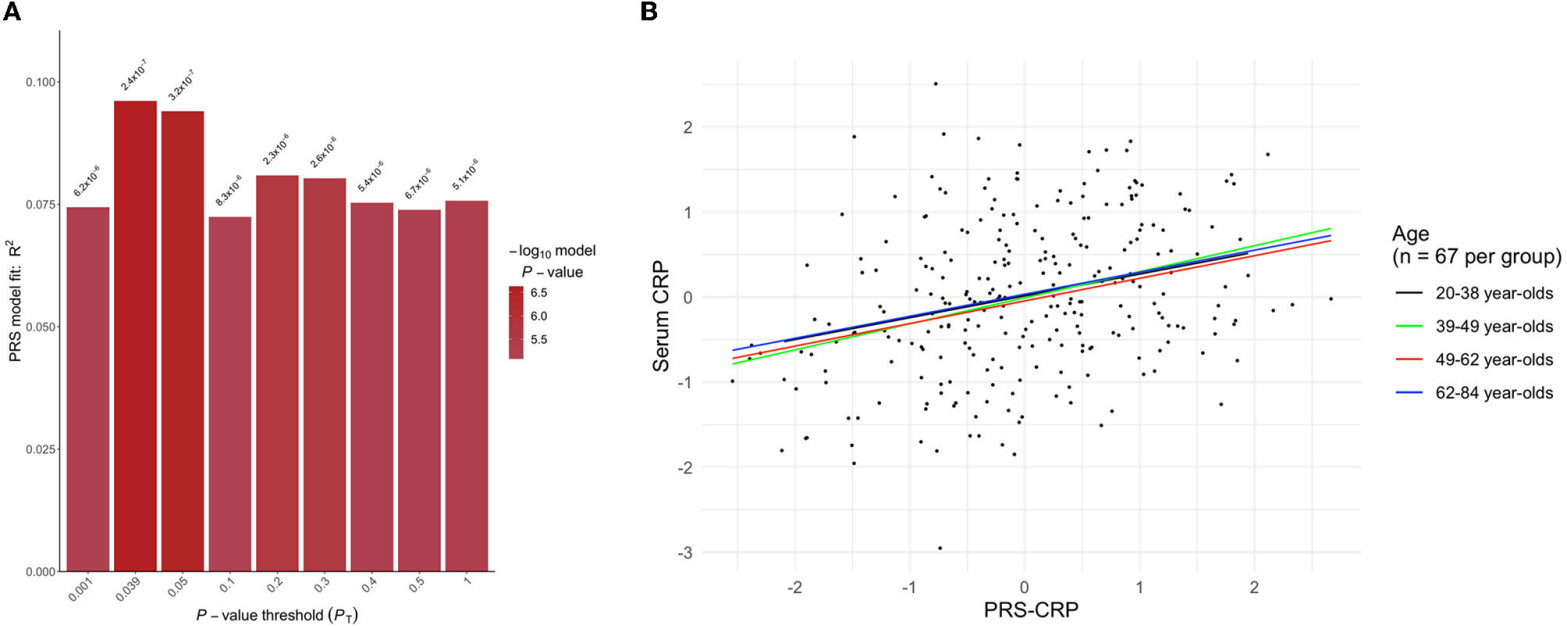
**(A)** Output from PRSice-2 displaying a range of *p*-value thresholds (PT) tested, including the optimal PT as shown in the tallest bar at threshold PT = 0.039, which explained 9.61% of the variance (*p* = 2.362 × 10–7). **(B)** A scatter plot showing the positive correlation between polygenic risk scores for C-reactive protein levels (PRS-CRP; adjusted for seven PCs) and serum CRP levels (pg mL^−1^; log transformed and adjusted for age, gender, BMI, assay run, smoking) stratified by age group.

In the independent SELCoH sample (*N* = 268, *M*_*age*_ = 49.82 years, 52.6% female; see Supplementary Table 3 for SELCoH participant characteristics), polygenic risk scoring revealed that the *p*-value threshold, *P*_*T*_ = 0.039 significantly predicted 9.61% of the variance in adjusted CRP levels (*p* = 2.362 × 10^−7^), (see Figure 1). This effect remained significant after correcting for the 500 thresholds tested (*p*_*adj*_ = 1.181 × 10^−4^). In the PROTECT sample, we applied the optimal threshold (*P*_*T*_ = 0.039) identified in the SELCoH sample to generate a PRS-CRP. This resulted in the inclusion of 51,118 SNPs after clumping. This PRS-CRP was then used in downstream analyses.

### 3.3. No relationship between PRS-CRP and cognitive function in whole sample

The results of the regression analyses in the whole sample with forced entry are summarized in Table 4. After adjusting for covariates, PRS-CRP was not associated with any of the cognitive outcomes in the whole sample (*p*_*s*_ > 0.074).

**Table 4:** Whole sample linear regression results of PRS-CRP on each cognitive outcome.

**Test battery Cognitive outcome**	**β**	***SE β***	***t***	***p***	***R^2^***
**PCTB**					
Paired associates learning score	0.00	0.01	0.52	0.606	< 0.001
Digit span test score	0.00	0.01	−0.12	0.908	< 0.001
Self–ordered search score	0.03	0.02	1.76	0.079	< 0.001
Verbal reasoning score	0.08	0.08	0.95	0.342	< 0.001
**CogTrack** ^*TM*^					
Delayed visual recognition accuracy (original image)	−0.05	0.08	−0.61	0.544	< 0.001
Delayed visual recognition accuracy (distractor image)	−0.21	0.18	−1.17	0.242	< 0.001
Simple Reaction Time Speed Median	−0.06	0.60	−0.11	0.915	< 0.001
Digit Vigilance Speed	−0.12	0.48	−0.25	0.803	< 0.001
Digit Vigilance Target Accuracy	−0.03	0.02	−1.25	0.213	< 0.001
Digit Vigilance False Positive Responses	0.00	0.01	−0.04	0.967	< 0.001
Choice Reaction Time Speed Median	0.09	0.57	0.16	0.873	< 0.001
Choice Reaction Time Accuracy	−0.04	0.02	−1.79	0.074	< 0.001

PCTB, PROTECT Cognitive Battery; PRS-CRP, polygenic risk scores for C-reactive protein; SE, Standard Error. Analyses are adjusted for age, sex, education, employment status, number of test sessions completed, and the first six ancestry principal components. Beta coefficients represent unit of increase in cognitive outcome per 1 standard deviation increase in PRS. *R*^2^ represents the proportion of variance explained by the PRS-CRP (i.e., *R*^2^ of the model with only covariates subtracted from the *R*^2^ of the full model).

### 3.4. PRS-CRP is positively associated with spatial working memory in the oldest adults only

**Figure 2:**
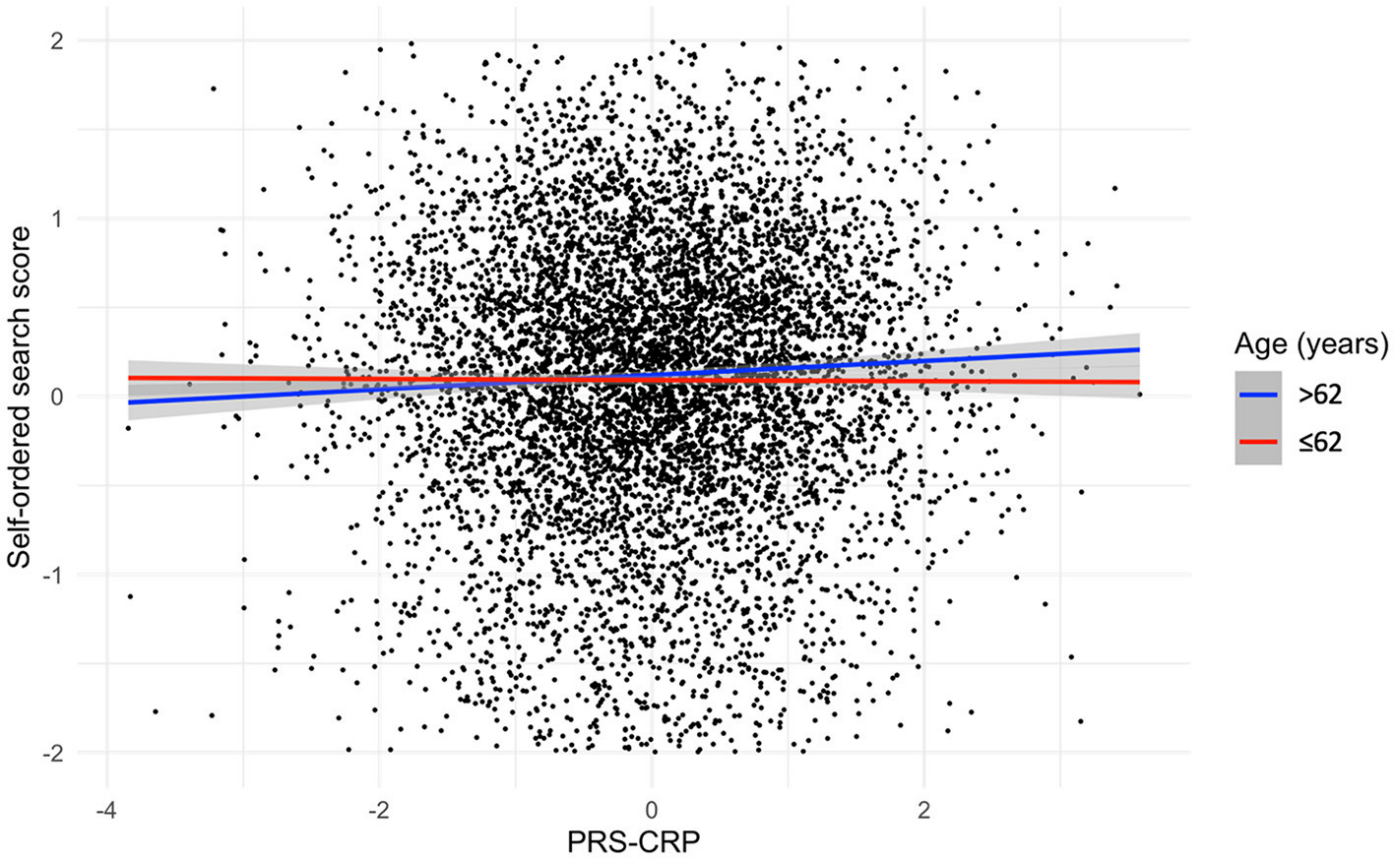
Relationship, split by median age, between PRS-CRP and self-ordered search score, adjusted for covariates (shaded areas are 95% confidence intervals).

To investigate whether inter-individual variation in CRP levels has a differential impact on older adults, we stratified our sample by median age (i.e., 62 years) and repeated our analysis. Table 5 summarizes the results of regression analyses adjusted for covariates with forced entry stratified by age. In older individuals, we observed a significant positive association between PRS-CRP and self-ordered search score (β = 0.08, *p* = 0.002, *R*^2^ = 0.003; Figure 2) that remained significant following correction (*P*_Bonferroni_ = 0.024). Additionally, higher PRS-CRP was nominally associated with better verbal reasoning (β = 0.28, *p* = 0.017, *R*^2^ = 0.001) and faster reaction time on the choice response task (β = −1.77, *p* = 0.034, *R*^2^ < 0.001) in older individuals. In younger individuals (i.e., 50–62 year olds), higher PRS-CRP was nominally associated with poorer accuracy on the digit vigilance (β = −0.07, *p* = 0.024, *R*^2^ < 0.001) and slower reaction time on the choice response task (β = 1.76, *p* = 0.023, *R*^2^ < 0.001). All other results were not significant at even the nominal level in both age groups (*p*_*s*_ > 0.146).

**Table 5:** Multiple regression results of PRS-CRP on each cognitive outcome, split by median age (62 years).

**Test battery**	**62 years and under**						**Over 62 years**					
**Cognitive outcome**	β	***SE** β*	***t***	***p***	***R** ^2^*	***n***	β	***SE** β*	***t***	***p***	***R** ^2^*	***n***
**PCTB**												
Paired associates learning score	0.00	0.01	−0.03	0.978	< 0.001	3,874	0.01	0.01	0.81	0.419	< 0.001	3,595
Digit span test score	−0.01	0.02	−0.49	0.626	< 0.001	3,931	0.01	0.02	0.42	0.677	< 0.001	3,622
Self–ordered search score	−0.01	0.03	−0.47	0.636	< 0.001	3,838	0.08	0.03	3.10	**0.002** ^*^	0.003	3,568
Verbal reasoning score	−0.11	0.12	−0.89	0.371	< 0.001	3,846	0.28	0.12	2.39	**0.017**	0.001	3,618
**CogTrack** ^*TM*^												
Delayed visual recognition accuracy (original image)	−0.14	0.11	−1.31	0.191	< 0.001	3,619	0.06	0.12	0.51	0.612	0.000	3,313
Delayed visual recognition accuracy (distractor image)	−0.22	0.24	−0.94	0.347	< 0.001	3,614	−0.14	0.27	−0.54	0.589	0.000	3,364
Simple reaction time speed median	0.86	0.82	1.06	0.290	< 0.001	3,610	−1.07	0.88	−1.21	0.225	0.000	3,349
Digit vigilance speed	0.38	0.65	0.58	0.561	< 0.001	3,679	−0.73	0.71	−1.02	0.307	0.000	3,402
Digit vigilance target accuracy	−0.07	0.03	−2.26	**0.024**	0.001	3,698	0.02	0.04	0.48	0.632	0.000	3,407
Digit vigilance false positive responses	0.01	0.02	0.70	0.481	< 0.001	3,654	−0.02	0.02	−0.82	0.411	0.000	3,336
Choice reaction time speed median	1.76	0.77	2.28	**0.023**	0.001	3,666	−1.77	0.84	−2.12	**0.034**	0.001	3,396
Choice reaction time accuracy	−0.03	0.03	−1.08	0.280	< 0.001	3,639	−0.04	0.03	−1.46	0.146	0.001	3,370

PCTB, Protect Cognitive Battery; PRS-CRP, polygenic risk scores for C-reactive protein; SE, Standard Error. Nominally significant results are in boldface (i.e., *p* < 0.05); ^*^indicate those that survived the Bonferroni corrected threshold (i.e., *p* < 0.004). Analyses are adjusted for age, sex, education, employment status, number of test sessions completed, and the first six ancestry principal components. Beta coefficients represent unit of increase in cognitive outcome per 1 standard deviation increase in PRS. *R*^2^ represents the proportion of variance explained by the PRS-CRP (i.e., *R*^2^ of the model with only covariates subtracted from the *R*^2^ of the full model).

### 3.5. Sensitivity analyses

To confirm that our PRS-CRP would predict CRP levels across the age ranges encompassed within PROTECT, we stratified the SELCoH sample by age and as part of a sensitivity analysis confirmed its predictive power, summarized in Supplementary Table 4. All associations were in the same direction (positive) across age groups suggesting that the PRS-CRP has predictive power across a range of adult ages, though there was a small descriptive difference in the amount of variance explained across groups (see, right panel of Figure 2 and Supplementary Table 4). The PRS-CRP explained similar amounts of variance across age groups tested, with the most variance explained in participants aged 39–49 years (β = 0.30, *p* = 0.007, *R*^2^ = 0.107), followed by 49–62 year-olds (β = 0.27, *p* = 0.016, *R*^2^ = 0.086), then 20–38 year-olds (β = 0.26, *p* = 0.017, *R*^2^ = 0.084), with the least variance explained in 62–84 year-olds (β = 0.24, *p* = 0.030, *R*^2^ = 0.070).

## 4. Discussion

To date, few studies have investigated the shared genetic relationship between CRP levels and cognitive function. We extended current research in this area by using a more powerful PRS-CRP and more comprehensive cognitive battery applied to a large sample of older adults. We did not observe any significant associations in the whole sample, even at the nominal level. Stratifying our sample by median age, in individuals aged 62 years and under, higher PRS-CRP was nominally associated with slower reaction times on the choice reaction task and lower accuracy on the digit vigilance task. Considering individuals aged over 62, higher PRS-CRP was nominally associated with better verbal reasoning and faster reaction times on the choice reaction task. Higher PRS-CRP was also significantly associated with better self-ordered search score in this older group (surviving correction for multiple testing).

Spatial working memory tasks, like the self-ordered search, are known to depend on the hippocampus (Spellman et al., 2015). Increased permeability of the blood-brain barrier with age may mean that CRP and inflammatory cytokines are more readily able to cross the blood-brain barrier to affect brain function (Erdö et al., 2016), and neurogenesis in the hippocampus (Ekdahl et al., 2003). This may explain why we observed effects in the older group only, albeit in the opposite direction to what we had hypothesized. That said, we did not find significant associations for all cognitive outcomes known to involve the hippocampus (i.e., the delayed visual recognition task).

When stratifying by median age we found some nominal effects in opposite directions (e.g., reaction time on the choice response task), which may mean that effects were masked when considering the sample overall. Our findings suggest that higher genetically predicted CRP levels may have positive effects in older individuals (>62 years) in specific aspects of cognition. This is in line with previous studies in adults over the age of 75 that found that increased CRP predicts better memory performance and decreased risk for cognitive decline (Silverman et al., 2009; Lima et al., 2014). An inverse relationship between CRP levels and onset of Alzheimer's disease has also been observed, whereby in adults >70.6 years, higher CRP was protective, but in adults aged 60–70.5 years it was adverse (e.g., Gabin et al., 2018). Perhaps relatedly, brain gene-expression studies have shown prominent changes in gene expression during the sixth to seventh decades, suggesting that this period is a critical transition point in brain aging (Berchtold et al., 2008) and that inflammatory genes are upregulated in brains of cognitively healthy older adults, but are down-regulated in dementia (Katsel et al., 2009). Others have suggested that a “protected survivor model” explains inverse age-dependent associations between CRP and cognitive aging, whereby the association of the risk factor with survival does not change within an individual, the association in the surviving population changes as its age increases due to differential mortality (e.g., Silverman and Schmeidler, 2018). Nonetheless, more research is needed to determine why higher peripheral levels of inflammation may have a protective role in healthy individuals ≥70 years. Our results and those of other studies highlight the importance of carefully considering the age of the sample in studies of (measured and genetically predicted) CRP and cognition in older adults.

The only previous study to our knowledge using a PRS-CRP to investigate the association between CRP levels and cognitive function in middle-older age adults (i.e., Milton et al., 2021), found a positive association between PRS-CRP and performance on the pairs matching test (visuospatial memory); though effect sizes were small. In this study, we did not find any associations between PRS-CRP and performance on the paired associates learning task (visuospatial memory). However, we did observe a statistically significant positive association between PRS-CRP and self-ordered search task performance (spatial working memory and executive function) in individuals aged over 62 years. Notably, both our results and those of Milton et al. suggest that higher genetically predicted CRP levels may be protective for spatial memory, contrary to hypotheses based on non-human animal work. Further, Milton et al. (2021) did not observe any associations between PRS-CRP and the reaction time test (processing speed). While we did not observe any associations between PRS-CRP and reaction times on the simple reaction time or digit vigilance task, we did observe a nominally significant association for reaction times on the choice reaction time task in analyses split by median age, with effects in opposite directions.

Together, our results suggest either the absence of a shared genetic relationship or a weak age-dependent and domain-specific genetic relationship between CRP and cognitive function in adults aged ≥50 years of European ancestry. Given the small effect sizes and limited significance of observed results, environmental factors experienced during the life course may better explain previous associations between CRP levels and cognitive function in middle-older aged adults than genetic factors. Indeed, prior infection has been linked not only to CRP levels but also to reduced cognitive function (e.g., Stebbins et al., 2021). Additionally, aside from pathogens, other more distal factors, such as pollution and traumatic events, have been linked to both elevated CRP levels and increased cognitive decline (Olvera Alvarez et al., 2018). It is also possible that higher peripheral inflammation negatively and preferentially impacts individuals already presenting with disease symptoms, or those at high risk of neuropsychiatric disease. For instance, the behavioral effects of LPS administration in rodents are modified by environment (e.g., stress; Barnum et al., 2012) and strain (e.g., Painsipp et al., 2008).

This current study had several limitations. Firstly, as is the case with many cognitive typical aging studies (see, Murman, 2015), the sample included an over-representation of women and those of a higher education level. While we adjusted for these variables in analyses, some caution is needed before generalizing findings to the wider population. The requirement for participants to have access to a computer and the internet could have further impacted the representativeness of the sample, particularly at older ages, where computer and internet use is less common (Niehaves and Plattfaut, 2014). Our results lack generalisability beyond European ancestry populations, namely because the GWAS of CRP levels from which the PRS was derived was based on individuals of European ancestry. Additionally, the ancestral composition of the cohort meant that we would have been underpowered to detect effects in other ancestral groups. Further research in more representative and diverse samples is therefore needed. Future research studies should collect blood for CRP assessment alongside biological material for genotyping, to ensure the effects of current CRP and genetic levels of CRP are accounted for when investigating the relationship between CRP and cognitive function. This would also facilitate drawing meaningful conclusions about environmental factors that may help to explain the observed phenotypic relationship between cognitive function and CRP levels. Mendelian randomization in larger samples of older individuals could also be used to confirm our mostly null findings.

## 5. Conclusion

Our results suggest either the absence of a shared genetic relationship or a weak positive relationship between CRP and working memory function in European adults aged ≥50 years. In light of prior research, the absence of a genetic relationship suggests environmental factors experienced during the life course might be affecting both CRP and cognitive measures, explaining previously reported associations.

## Data availability statement

The data analyzed in this study is subject to the following licenses/restrictions: This study was conducted using secondary data collected as part of the UK version of the PROTECT ongoing study. PROTECT data are available to investigators outside the PROTECT team after request and approval by the PROTECT Steering Committee. Requests to access these datasets should be directed to protect.data@exeter.ac.uk.

## Ethics statement

The studies involving human participants were reviewed and approved by London Bridge National Research Ethics Committee (reference: 13/LO/1578). The patients/participants provided their written informed consent to participate in this study.

## Author contributions

AC, CB, DA, AH, and BC were involved in the design, funding, sample collection, and/or assessments within the ongoing PROTECT study. RA was involved in processing the genotype data and quality control. DD and MM were involved in designing the current work and advising on analysis strategies. AP and TP were involved in designing, analyzing, and writing the manuscript. All authors provided edits and approved the manuscript prior to submission.
