# Supplementary material for: Limited evidence of a shared genetic relationship between C-reactive protein levels and cognitive function in older UK adults of European ancestry
Source: Front Dement. 2023 Aug 2;2:1093223. doi: 10.3389/frdem.2023.1093223 (PMC11285585; doi:10.3389/frdem.2023.1093223)
Supplement: Supplementary file 1 [file Table_1.DOCX]

Supplementary Material

1. **Supplementary Tables**

## Supplementary Table 1. Description of the neuropsychological test batteries and the derived outcome measures used in analyses.

| Test | Outcome measures | Description | Reference |
| --- | --- | --- | --- |
| *PROTECT Cognitive Test Battery (PCTB)* | | | |
| Paired associates learning (PAL) | Summary score | Boxes on screen, which are “opened” in a randomised order. One or more of the boxes contain a shape. Each shape is then presented in the middle of the screen and participants must identify which box the shape had been located in. If the participant makes an error, the boxes are opened in sequence again to remind the participant of the locations of the shape. The number of shapes per trial increases throughout the task making it increasingly difficult. Participants are given three attempts to successfully complete each level. The outcome measure was the average number of correct object-place associations (“paired associates”) in trials that were successfully completed. This task measures visual-spatial working memory and learning. | (Owen et al., 1993) |
| Digit span | Summary score | A sequence of numbers appears on the screen, one at a time. At the sound of the beep, users click the numbers in the same order. Each successful trial is followed by a new sequence that is one digit longer than the last and each unsuccessful trial is followed by a new sequence that is one digit shorter than the last. This task measures working memory. | (Huntley et al., 2017) |
| Self-ordered search | Summary score | A series of boxes are present on the screen; one of the boxes will contain a token. The participant selects each box until they locate the token. The token is then placed in another box and again the participant must locate it. Participants are informed that the diamond will never be in the same box twice. Higher scores are achieved through efficient location of the diamond. This task measures working memory. | (Owen et al., 1990) |
| Verbal reasoning | Summary score | A statement appears at the top of the screen, and two objects underneath. The patient’s task is to reason about the relationships among the objects and determine if the statement is true or false. Responding quickly and accurately is required for high scores. There is no set upper or lower limit as the participants can attempt as many trials as they can manage within a specific timeframe. The outcome measure was the total number of trials answered correctly in 90 seconds, minus the number answered incorrectly. Higher scores indicate better performance. This task assesses verbal reasoning. | (Baddeley, 1968) |
| *CogTrack^TM^* | | | |
| Delayed Visual Recognition (Picture Recognition) | Target picture accuracy (%)  Distractor picture  accuracy (%) | At the start of the battery 20 pictures of everyday scenes and objects are presented on the screen, at the rate of one picture every 3 seconds, for the participant to remember. The participant is instructed that the pictures will all be shown again later, mixed with very similar ones. At the end of the battery the 40 pictures (20 original, 20 similar ‘distractors’) are presented one at a time, the order being counterbalanced such that half of the original pictures are presented prior to the distractors, and half afterwards. For each picture, the participant has to indicate whether or not it was the precise picture shown earlier, as quickly and accurately as possible, pressing the right arrow key on the keyboard if it was, and the left arrow if it was not. Each picture remains on the screen until a response is made. | (Wesnes et al., 2017) |
| Simple Reaction Time | RT (median) | The participant is instructed to press the right arrow key on the keyboard as quickly as possible every time a right-facing arrow containing the word ‘YES’ is presented in the centre of the screen. The participant is informed that only this stimulus will be presented and that it will remain there until a response is made. Fifty stimuli are presented with random inter-stimulus interval between one and 3.5 seconds. The speed of each response is recorded. | Wesnes et al. (2017) |
| Digit vigilance | RT (median)  Accuracy (%)  False alarms (n) | A target digit from one to nine is randomly selected and constantly displayed on the right-hand side of the screen. A series of 450 digits is then presented one at a time in the centre of the screen at the rate of 150 per minute. The participant is required to press the right arrow key on the keyboard as quickly as possible every time a presented digit matches the target digit on the right. Correct detections, the speed of the detections and responses made in error (false alarms) are recorded. | Wesnes et al. (2017) |
| Choice Reaction Time | RT (median)  Accuracy (%) | There are two possible stimuli in this task that can appear on screen, either the right-facing arrow used in Simple Reaction Time, or a left facing version of the arrow, with the word ‘NO’ in the middle. One of these two stimuli are randomly displayed in the middle of the screen. The participant is required to respond by pressing the corresponding key as quickly and accurately as possible every time the stimuli appear on screen. The stimulus is presented until a response is made. There are 50 successive trials. The interval between trials varies randomly between one and 3.5 seconds. | Wesnes et al. (2017) |

*Note.* RT = reaction time.

**Supplementary Table 2.** Number and percentage of missing data points and outliers removed for each cognitive outcome.

| *Test battery* | Missing | | Outliers | |
| --- | --- | --- | --- | --- |
| Cognitive outcome | *n* | *%* | n | % |
| *PCTB (n = 7817)* |  |  |  |  |
| Paired associates learning | 0 | 0.00 | 348 | 4.45 |
| Digit span test | 0 | 0.00 | 264 | 3.38 |
| Self-ordered search | 1 | 0.01 | 410 | 5.24 |
| Verbal reasoning | 0 | 0.00 | 353 | 4.52 |
| *CogTrack^TM^ (n = 7275)* |  |  |  |  |
| Delayed visual recognition accuracy (original image) | 13 | 0.00 | 330 | 4.54 |
| Delayed visual recognition accuracy (distractor image) | 13 | 0.00 | 284 | 3.90 |
| Simple Reaction Time Speed Median | 0 | 0.01 | 316 | 4.34 |
| Digit Vigilance Speed | 1 | 0.00 | 193 | 2.65 |
| Digit Vigilance Target Accuracy | 0 | 0.00 | 170 | 2.34 |
| Digit Vigilance False Positive Responses | 0 | 0.18 | 285 | 3.92 |
| Choice Reaction Time (median) | 0 | 0.18 | 213 | 2.93 |
| Choice Reaction Time Accuracy | 0 | 0.00 | 266 | 3.66 |

*Note.* PCTB = PROTECT cognitive test battery. Outliers +/- 2 standard deviations from the mean (adjusting for covariates) were removed prior to regression analyses.

**Supplementary Table 3.** SELCoH Participant characteristics (*N* = 268)

| Variable | *M* |
| --- | --- |
| Age (mean (SD)) | 49.82 (0.91) |
| Sex (n, %) |  |
| Male | 124, 47.4% |
| Female | 141, 52.6% |
| BMI (mean (SD)) | 26.52 (5.19) |
| Smoking (n, %) |  |
| Never smoked | 89, 33.2% |
| Currently smoking | 56, 20.90% |
| Previously smoked | 123, 45.9% |
| Serum CRP, mg/dL (mean (SD)) | 0.373 (0.544) |

## *Note.* SELCoH = South East London Community Health Study; SD = standard deviation.

## Supplementary Table 4. Multiple regression results of PRS-CRP on CRP level in the SELCoH sample split by age (*ns* = 67).

| Age group | *β* | *SE β* | *t* | *p* | *R^2^* |
| --- | --- | --- | --- | --- | --- |
| 20-38 years | 0.26 | 0.11 | 2.44 | .017 | 0.084 |
| 39-49 years | 0.30 | 0.11 | 2.80 | .007 | 0.107 |
| 49-62 years | 0.27 | 0.11 | 2.48 | .016 | 0.086 |
| 62-84 years | 0.24 | 0.11 | 2.21 | .030 | 0.070 |

*Note.* PRS-CRP = polygenic risk scores for C-reactive protein; CRP = C-reactive protein; SE = Standard Error. Significant results are in boldface (i.e., *p* <.05). CRP levels were adjusted for age, gender, BMI, assay run, smoking and log transformed prior to analyses. The first seven ancestry principal components (PCs) were included as covariates. *R^2^* represents the proportion of variance explained by the PRS-CRP (i.e., *R^2^* of the model with only covariates (i.e., seven PCs) subtracted from the *R^2^* of the full model).
